# The role of endocrine-disrupting chemicals in obesity: mechanisms, evidence, and public health implications

**DOI:** 10.3389/fendo.2026.1782328

**Published:** 2026-04-14

**Authors:** Viridiana M. Mendoza-Martínez, Aldo González, Lucía A. Méndez-García, Galileo Escobedo, Miguel A. Fonseca-Sánchez, Arturo Cérbulo-Vázquez, Marcela Esquivel-Velázquez, Guillermo Meléndez-Mier, Nallely Bueno-Hernández

**Affiliations:** 1 Proteomics and Metabolomics Laboratory, Research Division, Hospital General de México Dr. Eduardo Liceaga, Secretaría de Salud, Mexico City, Mexico; 2 Escuela de Dietética y Nutrición del ISSSTE, Mexico City, Mexico; 3 Facultad de Salud Pública y Nutrición, Universidad Autónoma de Monterrey, Monterrey, Mexico

**Keywords:** adipogenesis, endocrine disruptors, environmental exposure, epigenetics, obesity, public health

## Abstract

**Introduction:**

Obesity is increasingly recognized as a multifactorial disease influenced by environmental exposures, including endocrine-disrupting chemicals (EDCs). These substances interfere with hormonal signaling and may contribute to adipogenesis and metabolic dysfunction.

**Methods:**

This narrative mini-review drew on epidemiological, experimental, and clinical research and examined the most recent literature on the role of EDCs in adipogenesis. It focuses on the mechanisms of action.

**Results:**

Studies report that a wide variety of EDCs affect adipogenic differentiation through pathways mediated by nuclear receptors (PPARγ/RXR) and through broader endocrine disruptions involving estrogen-, glucocorticoid-, and thyroid-related pathways. Adiposity, including body weight, BMI, and abdominal obesity, is associated with chemical exposure, according to clinical data from human studies. Since they may differ based on the EDCs, the timing of exposure, and sex, the strength and consistency of the effects are still not entirely understood.

**Conclusions:**

One important environmental factor in the development of obesity is EDCs. Public health interventions should prioritize identifying and regulating them.

## 1 Introduction

Overweight and obesity have been considered the pandemic of the 21st century, characterized by excessive accumulation of adipose tissue, generally associated with high energy intake and low physical activity (1). However, there is growing interest in less conventional environmental factors, such as exposure to chemicals in everyday products, which can interfere with hormonal function. These substances, known as endocrine disruptors (EDCs), are found in numerous industrial and household materials and were initially studied for their impact on reproduction by altering steroid and thyroid hormone levels. Currently, it is recognized that some of these compounds also affect metabolic processes, such as adipocyte function, promoting imbalances in energy metabolism and contributing to the development of obesity (2).

Constant exposure to these substances in industrialized contexts, along with their persistence and bioaccumulation, has generated growing concern about their possible role in the rise in metabolic diseases. Some EDCs have been termed obesogens because they interfere with the pathways that regulate adipogenesis and body weight control, contributing not only to overweight and obesity but also to other associated metabolic disorders (3).

Critical windows of vulnerability have been identified, especially during pregnancy, in which exposure to these compounds can induce subtle epigenetic modifications. These changes may remain silent during childhood and manifest later, in adolescence or adulthood, with increases in body weight, body mass index (BMI), and waist circumference (4).

These long-term effects on metabolic programming laid the foundation for the concept of “obesogens,” formally proposed in 2006 by Bruce Blumberg, which has gained relevance in the last decade as a complementary explanatory model to traditional factors of obesity. Beyond adiposity, EDCs have been linked to the development of metabolic syndrome, type 2 diabetes, and non-alcoholic fatty liver disease (5).

Despite the multifactorial origin of the metabolic disease epidemic, recent data indicate that environmental factors, specifically exposure to EDCs, play a significant role. Investigating the effects of these substances on adipose tissue differentiation and function is crucial. According to scientific data, one important way that people are exposed to EDCs is through their diet. These substances can migrate from packaging materials, particularly plastics and canned goods, or enter food through pesticides and fertilizers (6). A 2013–2016 NHANES analysis showed that even following dietary patterns such as the Mediterranean diet or high scores on the Healthy Eating Index did not reduce exposure to non-persistent EDCs. In fact, some diets were associated with higher urinary levels of nitrate and perchlorate, compounds known to interfere with thyroid hormone production (7).

Obesity, characterized by the accumulation of adipose tissue, affects various biological processes. Weight gain mediated by this accumulation, as well as the location of the fat, which can be visceral, is reflected in changes in waist circumference, providing indirect information about central adiposity. Evidence suggests that EDCs can alter adipose tissue function, fat distribution, and metabolic efficiency, even in the absence of increased BMI (1).

With this mini narrative review, we aim to integrate recent experimental and human evidence linking exposure to EDCs with the development of obesity and adipogenesis. The review focuses on biological plausibility and summarizes the main hormonal and nuclear receptor-mediated mechanisms described in *in vitro* studies and animal models. We concentrate on developmental susceptibility, sex-specific metabolic responses, and emerging data on combined environmental exposures, with the goal of contextualizing the current evidence and highlighting areas where knowledge remains incomplete.

## 2 Materials and methods

A non-systematic literature search in PubMed and Scopus served as the foundation for this narrative mini-review. Priority was given to peer-reviewed papers released between January 2015 and the beginning of 2025. Combinations of “endocrine-disrupting chemicals,” “obesogens,” “adipogenesis,” “obesity,” “body mass index,” “visceral fat,” and “epigenetics” were among the search terms used.

Included were observational or clinical studies conducted on humans as well as experimental studies (*in vitro* and animal models). When they pertained to the definition of key terms, biological processes, or the evolution of the obesogen hypothesis over time, older or foundational studies were carefully included.

Relevance to biological plausibility, developmental susceptibility, nuclear receptor-mediated mechanisms, and new evidence from human populations, such as studies addressing mixture exposures and sex-specific metabolic responses, was considered when selecting studies. In keeping with the narrative focus of this Mini Review, neither a formal quality scoring nor a meta-analysis was conducted.

## 3 Endocrine disruptors: definition, classification, and sources of exposure

EDCs, also known as hormone-disrupting chemicals, can be natural or synthetic compounds that mimic, block, or interfere with normal hormonal function in human and animal organisms, causing excessive or inappropriate agonist responses, or as hormonal antagonists, blocking receptors, altering the synthesis, metabolism, or transport of hormones (8).

The Endocrine Society has defined EDCs as “substances in our environment, foods, and consumer products that interfere with the biosynthesis, metabolism, or action of hormones, resulting in a deviation from normal homeostatic control or reproduction” (9). On the other hand, the Food and Drug Administration (FDA) defines EDCs as “chemicals that interfere with endocrine systems and cause adverse effects” (10). This means that EDCs can induce epigenetic modifications in hormone-producing or hormone-responsive tissues, disrupt hormone synthesis, alter transport across cell membranes, and alter hormone distribution or circulating concentrations (11).

There are approximately 85,000 chemicals registered in the industry, with little individual toxicological research. It is estimated that approximately 1,000 meet the criteria for EDCs (**Table 1**). These compounds are present in a wide range of consumer products, including food packaging, construction materials, pesticides, clothing, upholstery, personal care products, detergents, cleaning agents, thermal paper, plastics, and medical equipment (12).

**Table 1:** Main endocrine-disrupting chemical substances.

Substance group	Disrupt
Synthetic hormones	Ethinylestradiol
Plastics	Bisphenol phthalates (BPA)
Pesticides and fungicides	Organotins, Methoxychlor, Chlorpyrifos, Dichlorodiphenyltrichloroethane (DDT), Vinclozolin
Solvents	Polychlorinated biphenyls (PCBs), Polybrominated biphenyls (PBBs), Dioxins
Personal care products	Triclosan
Phytoestrogens	Genistein, Coumestrol

Exposure routes are diverse, with oral, dermal, and inhalation being the most common, as well as subcutaneous and intravenous routes (4). The evidence of the EDCs reported to affect reproductive function in both men and women and have been associated with higher levels of adiposity and blood pressure in the pediatric population, where early exposure to EDCs is a determining factor for the development of future complications (13).

## 4 Linking EDCs to adipogenesis

It appears that individuals exposed to obesogens are more susceptible to increased body fat, even when following a diet and exercise regimen. This may be related to the fact that these compounds promote the differentiation of new adipocytes. These effects have been demonstrated primarily in experimental models, in which obesogens can alter basal metabolic regulation, interact with the gut microbiota, and modify hormonal pathways regulating appetite and satiety (1, 4).

An example of this effect was reported by Li et al. in 2012, who identified triflumizole (TFZ), an imidazole fungicide used in agriculture, as an obesogen that acts by activating the peroxisome proliferator-activated receptor gamma (PPARγ). In this study, TFZ induced adipogenesis in human mesenchymal stem cells and murine 3T3-L1 preadipocytes, even at nanomolar concentrations. Increased adipogenic gene expression and decreased osteogenic gene expression were also observed in an *in vitro* model, indicating a redirection of cell fate toward the adipocyte lineage (14).

### 4.1 Nuclear receptor–mediated mechanisms, *in vitro* and animal evidence

EDCs increase the body’s capacity to form adipocytes and accumulate lipids within them. Experimental studies have shown that they activate PPARγ, which regulates adipogenesis and the function of mature adipocytes, and facilitate the maturation of mesenchymal stem cells into adipocytes (15). PPARγ is the central transcriptional regulator of adipogenesis and a key molecular target of various obesogens. Its activation promotes the expression of genes involved in lipid metabolism and fat storage. This receptor forms heterodimers with RXR (9-cis retinoic acid receptor) and, upon ligand binding, modulates the expression of key genes involved in lipid storage, transport, and metabolism. Its target genes include fibroblast growth factor 1 (FGF1), G protein-coupled receptor 81 (GPR81), and adiponectin (16).

Reports on genistein, a phytoestrogen and possible EDCs (**Table 1**), have shown that it can induce adipocyte differentiation, lipid accumulation, and overexpression of adipogenic genes such as C/EBPα, C/EBPβ, and PPARγ *in vitro*, and promote adipogenesis in animal models by activating the PPARγ-RXR pathway (17).

Evidence for these mechanisms comes primarily from *in vitro* and animal studies, where compounds such as tributyltin (TBT) have been shown to bind and activate the PPARγ–RXR heterodimer, predisposing mesenchymal stem cells to adipogenic differentiation (18). In an *in vitro* study by Jie et al. they observed that in human macrophages, nanomolar concentrations of TBT strongly activated PPARγ, leading to increased intracellular lipid accumulation and overexpression of PPARγ target genes involved in lipid metabolism. These effects were attenuated in PPARγ-deficient macrophages, confirming receptor-specific mediation. In the same study, they observed *in vivo* that subchronic exposure to TBT at doses below the no-adverse-effect level also promoted PPARγ signaling and increased body weight in mice (19).

### 4.2 Other receptors involved in obesogenic effects of EDCs

Although the PPARγ receptor is considered a central regulator of adipogenesis, it is not the only molecular target of EDCs. Studies have shown that endogenous estrogens can bind directly to the estrogen receptor alpha (ERα) and to a wide variety of structurally diverse EDCs. In silico and experimental screening studies have demonstrated that various compounds can interact with the ligand-binding domain of ERα, leading to receptor agonism or antagonism and promoting transcriptional dysregulation. Therefore, the role of ERα as a critical molecule through which EDCs can alter adipocyte differentiation, lipid metabolism, and energy homeostasis is supported (20).

Another example is that chronic exposure to a low-dose pesticide mixture at NOAEL levels induces sustained activation of PXR and CAR, impacting the transcriptional reprogramming of glucose and lipid metabolism, without affecting body weight or adiposity. Using wild-type and receptor-deficient murine models, Dauwe et al. demonstrated that PXR activation alters glycemic response and insulin sensitivity via PI3K/Akt signaling pathways, whereas CAR deficiency alters circulating lipid profiles. This highlights the receptor-specific and genotype-dependent metabolic effect. It is suggested that exposure to these EDCs may promote subtle receptor-mediated alterations in metabolic signaling rather than an overt expansion of adipose tissue, thereby providing a mechanistic link between environmental exposures and early metabolic dysfunction (21).

Similarly, Lukowicz et al. exposed male and female mice to a dietary mixture of six commonly used pesticides at tolerable daily intake levels for 52 weeks. CAR receptor activation was shown to mediate sex-specific metabolic outcomes, with wild-type male mice exhibiting increased adiposity and body weight, as well as glucose intolerance and hepatic steatosis, while these effects were eliminated in CAR-deficient males. This demonstrates a causal role for CAR in pesticide-induced metabolic disturbance. On the other hand, wild-type females exhibited fasting hyperglycemia, oxidative stress, and metabolomic alterations in the absence of overt obesity, while CAR deficiency led to increased body weight and mortality. These findings show that EDCs can induce sexually dimorphic metabolic dysfunction through nuclear receptor-dependent mechanisms that precede or occur independently of overt adipose tissue expansion (22).

These sex-specific metabolic responses to endocrine-disrupting chemicals are not accidental but reflect an intrinsic sexual dimorphism in adipose tissue biology. Adipose tissue differs between men and women in terms of distribution, cellular composition, endocrine function, and inflammatory profile, all of which modulate susceptibility to metabolic disruption. Consequently, environmental exposures can exacerbate or attenuate adipogenic and metabolic outcomes in a sex-dependent manner, even in the absence of obesity (23).

## 5 Evidence from human studies on EDCs exposure and adiposity

Several epidemiological and experimental studies have examined how exposure to EDCs can promote adiposity development through hormonal, epigenetic, and metabolic mechanisms. This evidence supports the hypothesis that these substances alter adipose tissue programming, even from the early stages of development.

In 2018, Cardenas et al. evaluated the association between perfluoroalkyl and polyfluoroalkyl compounds (PFAS) and increased adiposity in adults at risk for type 2 diabetes, as well as the possible attenuating effect of lifestyle intervention. In this prospective study of 957 participants in the Diabetes Prevention Program (DPP), significant associations were observed between plasma PFAS concentrations and increased weight and hip circumference in the placebo group, comprising 476 individuals who did not receive an intensive diet or exercise intervention. For each doubling of total PFAS concentration, there was an average increase of 1.80 kg (95% CI: 0.43–3.17; p = 0.01) in weight and 1.03 cm (95% CI: 0.18–1.88; p = 0.02) in hip circumference at 9 years. These associations were not significant in the intensive intervention group, suggesting that diet and exercise may mitigate the obesogenic effects of these compounds (24).

On the other hand, in a randomized clinical trial (POUNDS Lost), Liu et al. (2018) evaluated the association between exposure to perfluoroalkyl substances (PFAS) and changes in body weight and basal metabolic rate (BMR) among people with obesity/overweight or participating in a 2-year weight-loss program. Baseline PFAS concentrations were not associated with initial weight loss; however, elevated levels of PFOS, PFOA, PFHxS, PFNA, and PFDA were significantly associated with greater weight regain between 6 and 24 months, particularly in women, who regained 1.7 to 2.2 kg more compared to those with lower PFAS levels. A more pronounced decline in RMR was documented during the weight-loss phase, and a smaller subsequent increase during the recovery phase, among those with higher plasma concentrations of these substances. These findings suggest that PFAS exposure may interfere with human body weight regulation, possibly through alterations in energy metabolism and hormonal mechanisms, highlighting the importance of considering environmental factors in the etiology of obesity (25). These alterations could be mediated by hormonal or epigenetic changes that affect basal metabolism and long-term energy efficiency.

Other studies have addressed simultaneous exposure to multiple pollutants with obesogenic potential, considering that actual exposure occurs in combination. Zhang et al. (26) evaluated the association between simultaneous exposure to a mixture of seven environmental compounds, including phenols, pesticides, and phthalate metabolites, and obesity in a representative sample of US adults (NHANES 2013–2014, n=1269). Using three complementary statistical models (generalized linear regression, WQS, and BKMR), they identified a significant relationship between co-exposure and overall (OR = 1.63; 95% CI: 1.21–2.20) and abdominal (OR = 1.66; 95% CI: 1.18–2.34) obesity. MCOP, BPA, and BPS were the compounds most consistently associated with increased risk. The compounds most consistently associated with increased obesity risk were MCOP, BPA, and BPS. The BKMR model showed a positive relationship between co-exposure to these pollutants and obesity, especially when exposure levels exceed the 60th percentile, suggesting a synergistic interaction between these compounds and adipogenesis (26).

Data from the 2013–2016 NHANES survey (n = 7780 adults) examined urinary phthalate metabolites (EPCs) in relation to sex hormones and obesity and reported that combined exposure to several metabolites, especially MCNP, MCOP, and MCPP, was associated with a higher likelihood of obesity (OR = 1.082 [CI: 1.027–1.141], p < 0.001). Furthermore, total testosterone and SHBG levels showed an inverse relationship, while estradiol levels increased with higher phthalate levels (27).

In addition to chemical compounds present in consumer products, air pollution has been documented to exert potential obesogenic effects. In a 2021 study, Bowe et al. analyzed the association between exposure to ambient fine particulate matter (PM2.5) and the risk of weight gain and obesity in a national cohort of 3,902,440 US veterans followed for a median of 8.1 years. The results showed that an average annual increase of 10 μg/m³ in PM2.5 levels was associated with an 8% increased risk of developing obesity (HR = 1.08; 95% CI: 1.06–1.11) and a 7% increased risk of gaining ≥4.54 kg (HR = 1.07; 95% CI: 1.06–1.08). Furthermore, significant intraindividual increases were observed in body mass index (0.140 kg/m² per year; 95% CI: 0.139–0.142) and body weight (0.968 lb. per year; 95% CI: 0.955–0.981). These results indicate that even low levels of air pollutants can promote adiposity development, possibly through hormonal and epigenetic mechanisms that are not yet fully understood (**Table 2**) (28).

**Table 2:** Evidence from human studies on the association between exposure to endocrine-disrupting chemicals and indicators of adiposity.

Author	Year	Study	Population	Country	Disruptor	How was the disruptor measured?	Visceral fat	BMI	Weight	Waist circumference
		Design			Evaluated					
Bowe et al. (28)	2021	Observational cohort	3,902,440 U.S. military veterans	United States	PM2.5 (particulate matter ≤2.5μm)	Geocoded satellite estimation by residence	Not reported	+0.140 kg/m²/year (95% CI: 0.139–0.142)	+0.968 lb/year (95% CI: 0.955–0.981)	Not reported
Cardenas et al. (24)	2018	Prospective study	957 adults at risk of T2DM (DPP)	United States	PFAS (perfluorinated compounds)	Plasma concentrations	Not reported	Not reported	↑1.80 kg (95% CI: 0.43–3.17) in the placebo group (no diet or exercise intervention)	↑1.03 cm (95% CI: 0.18–1.88) in the placebo group (no diet or exercise intervention)
Liu et al. (25)	2018	Randomized clinical trial	621 with overweight/obesity	United States	PFOS, PFOA, PFNA, PFHxS, PFDA	Plasma concentration (LC-MS/MS)	Baseline levels of PFOA and PFHxS were associated with a greater increase in visceral fat mass (r_s_ = 0.30 and 0.27, respectively; both P < 0.05)	Not reported	↑2.7 kg on average (6–24 months)	Not reported
Zhang et al. (26)	2019	Cross-sectional (NHANES)	1,269 adults	United States	Mixture of phenols, phthalates, pesticides (BPA, BPS, MCOP, MECPP, MEP, etc.)	Urinary levels measured	Abdominal obesity (58%)	Association with MCOP, BPA, BPS	Not reported	Positive association with MCOP, BPA, BPS

PM2.5 refers to particulate matter with a diameter of ≤2.5 micrometers. PFAS stands for per- and polyfluoroalkyl substances, including specific compounds such as PFOA (perfluorooctanoic acid), PFOS (perfluorooctane sulfonate), PFNA (perfluorononanoic acid), PFHxS (perfluorohexane sulfonic acid), and PFDA (perfluorodecanoic acid). BMI stands for Body Mass Index. T2DM refers to Type 2 Diabetes Mellitus, and DPP stands for the Diabetes Prevention Program. LC-MS/MS means Liquid Chromatography–Tandem Mass Spectrometry. BPA (Bisphenol A) and BPS (Bisphenol S) are phenols commonly used in the production of plastics. MCOP is mono(carboxyisooctyl) phthalate, MECPP is mono(2-ethyl-5-carboxypentyl) phthalate, and MEP is monoethyl phthalate. NHANES refers to the National Health and Nutrition Examination Survey. CI stands for Confidence Interval. r_s_ represents Spearman’s correlation coefficient.

EDCs have been shown to alter hormonal signaling by activating estrogen receptors, thereby promoting human adipogenesis even in the absence of glucocorticoid exposure. BPA, for example, increases the expression of enzymes involved in lipid accumulation and adipocyte differentiation by acting as a glucocorticoid receptor agonist (16, 29, 30). Furthermore, it has been documented that certain EDCs exhibit antithyroid activity, thereby preventing the synthesis of thyroid hormones necessary for maintaining basal metabolism (31).

Obesogens have been identified as agonists or antagonists of estrogen, androgen, and thyroid hormone receptors, altering endocrine signaling through multiple mechanisms. They can inhibit or stimulate hormone production, alter hormone metabolism, or alter hormone transport to target tissues. Although their effects occur at low doses, some EDCs bioaccumulate in tissues (4). A 2023 study assessed the association between bisphenol A (BPA) exposure and thyroid cancer risk in patients with nodular goiter, as reported by Marotta et al. This study, which involved 96 patients, demonstrated that although BPA exposure was not significantly linked to thyroid cancer in subjects of normal weight, those who were overweight or obese had a significantly higher risk of the disease (OR: 5.3; p = 0.028). This association was independent of factors such as smoking, alcohol, occupational exposure, and phthalates, although it was attenuated after adjusting for metabolic syndrome. Furthermore, overweight/obese patients exposed to BPA had higher thyroid-stimulating hormone (TSH) levels, reinforcing the hypothesis that BPA may alter the thyroid axis through hormonal mechanisms, especially in individuals with greater adiposity (32).

## 6 Emerging ideas, research gaps, and future perspectives

Recent advances in this field have broadened the scope of the hypothesis that obesity is a multifactorial pathology, in which no single factor can be considered. This necessitates more comprehensive frameworks that account for the interplay among environmental exposures, gender-specific metabolic responses, and critical windows of vulnerability. Even without manifest obesity, low-dose combinations of EDCs can act through multiple nuclear receptors and induce mild metabolic reprogramming, according to new experimental and epidemiological analyses. This underscores the importance of employing techniques that assess metabolism using both mixtures and unconventional standards. Despite these advances, significant gaps in research remain.

Human studies are still limited by their reliance on single measurements of non-persistent chemicals, residual confounding factors, and the difficulty in establishing causal inferences. Furthermore, their measurements are often based on BMI, paying less attention to indicators such as visceral fat, ectopic fat deposition, and metabolic efficiency. Longitudinal designs with repeated exposure assessments, exposome-based approaches, and advanced statistical models capable of capturing mixing effects and nonlinear dose-response relationships should be the focus of future research.

It is crucial to focus more on developmental chronology, sex-specific vulnerability, and mechanistic biomarkers that link exposure to metabolic outcomes to support causal inferences.

## 7 Conclusion

Exposure to EDCs is highly variable and is often not considered in clinical histories or conventional epidemiological studies. Currently, the actual number of exposed individuals is unknown, and there are no standardized ranges for classifying exposure levels. Furthermore, the multiple routes of entry into the body, including not only diet but also inhalation and dermal contact, further complicate the quantification and accurate assessment of its effects. Evidence from animal models suggests that EDCs may play a significant role in regulating energy metabolism, adipocyte differentiation and maturation, and the accumulation of fat tissue, as shown in the graphical abstract. However, further human studies are required to confirm and characterize this association. Given its potential to directly activate metabolic pathways involved in adipogenesis, exposure to EDCs could constitute a key environmental factor in the development of overweight and obesity and therefore deserves greater attention in clinical, preventive, and public health settings.

## Glossary

BMI: Body Mass Index
BPA: Bisphenol A
BPS: Bisphenol S
CI: Confidence Interval
DPP: Diabetes Prevention Program
EDCs: Endocrine-Disrupting Chemicals
EDKB: Endocrine Disruptor Knowledge Base
EPA/USEPA: United States Environmental Protection Agency
FDA: Food and Drug Administration
HR: Hazard Ratio
NHANES: National Health and Nutrition Examination Survey
OR: Odds Ratio
PFAS: Per- and Polyfluoroalkyl Substances
PFOA: Perfluorooctanoic Acid
PFOS: Perfluorooctane Sulfonate
PFNA: Perfluorononanoic Acid
PFHxS: Perfluorohexane Sulfonic Acid
PFDA: Perfluorodecanoic Acid
RMR: Resting Metabolic Rate
RXR: Retinoid X Receptor
TFZ: Triflumizole
TSH: Thyroid-Stimulating Hormone
BKMR: Bayesian Kernel Machine Regression
WQS: Weighted Quantile Sum
MCOP: Mono(carboxyisooctyl) Phthalate
MCNP: Monocarboxiononil
MCPP: Mono(3-carboxipropil)
MECPP: Mono(2-ethyl-5-carboxypentyl) Phthalate
MEP: Monoethyl Phthalate
PM2.5: Particulate Matter ≤ 2.5 micrometers
SHBG: Sex hormone-binding globulin
TBT: Tributyltin.
